# 4-Des­oxy-4β-(4-methoxy­carbonyl-1,2,3-triazol-1-yl)podophyllotoxin dichloro­methane solvate

**DOI:** 10.1107/S1600536809050612

**Published:** 2009-11-28

**Authors:** Song Zuo, Hong Chen, Yanling Lu, Bo Cao, Dailin Liu

**Affiliations:** a School of Pharmaceutical Sciences, Tianjin Medical University, Tianjin 300071, People’s Republic of China; b Room of Pharmacognosy, Medical College of Chinese People’s Armed Police Forces, Tianjin 300162, People’s Republic of China; c Tianjin Key Laboratory for Biomarkers of Occupational and Environmental Hazards, Tianjin 300162, People’s Republic of China

## Abstract

In the title compound {systematic name: methyl 1-[12-oxo-10-(3,4,5-trimethoxy­phen­yl)-4,6,13-trioxa­tetra­cyclo­[7.7.0.0^3,7^.0^11,15^]hexa­deca-1,3(7),8-trien-16-yl]-1*H*-1,2,3-triazole-4-carboxyl­ate dichloro­methane solvate}, C_26_H_25_N_3_O_9_·CH_2_Cl_2_, the tetra­hydro­furan ring and the six-membered ring fused to it both display envelope conformations.

## Related literature

For similar structures of 4β-*N*-substituted-4-desoxypodo­phyllotoxin and derivatives, see: Bilal *et al.* (2008 ▶); Yu & Chen (2008 ▶); Van Maanen *et al.* (1988 ▶). For a review of the structures of azides and triazides, see: Bräse *et al.* (2005 ▶). For additional background to 1,3-dipolar azide–alkyne cycloaddition reactions, see: Hainsworth *et al.* (1985 ▶); Huisgen (1963 ▶); Jacobsen *et al.* (1988 ▶); Lee (2004 ▶). 
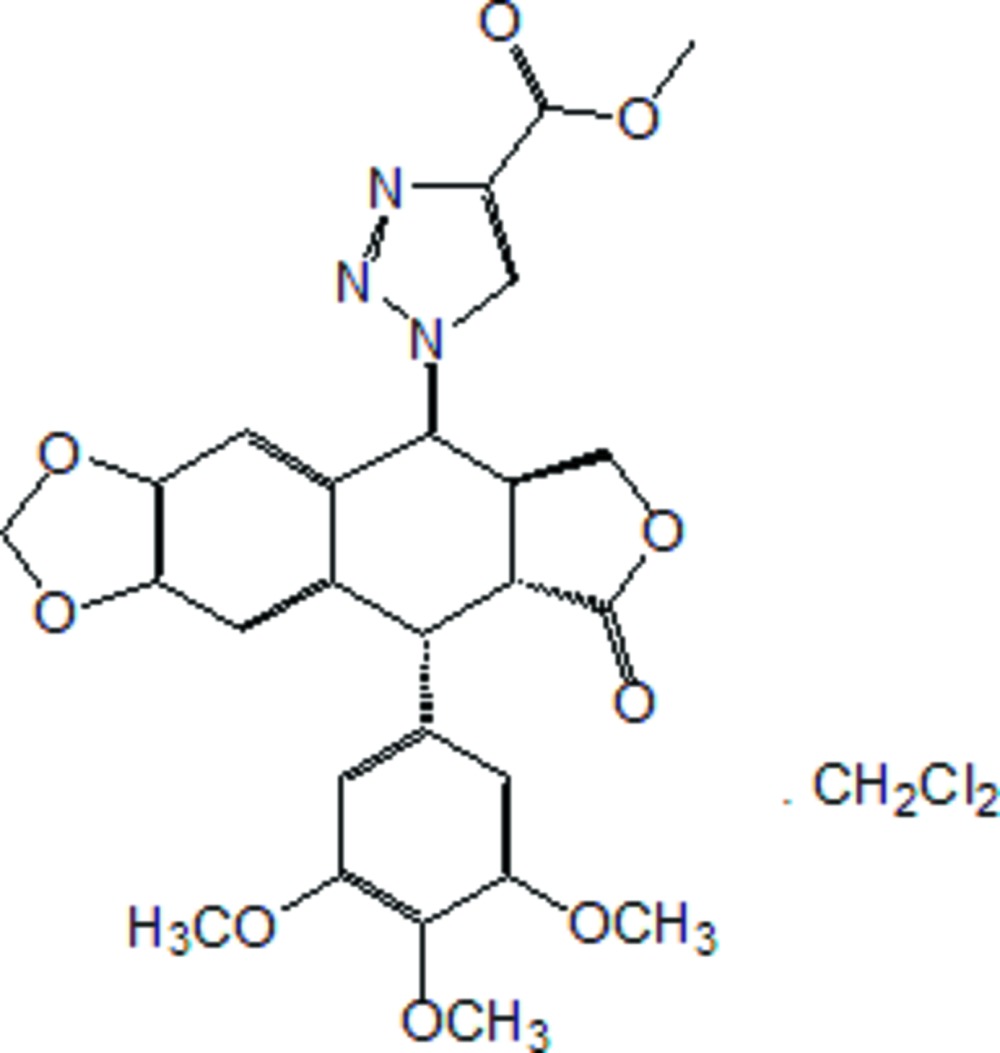


## Experimental

### 

#### Crystal data

C_26_H_25_N_3_O_9_·CH_2_Cl_2_
*M* *_r_* = 608.42
Orthorhombic, 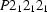

*a* = 10.377 (2) Å
*b* = 12.639 (3) Å
*c* = 20.463 (4) Å
*V* = 2683.9 (10) Å^3^
*Z* = 4
Mo *K*α radiation
μ = 0.30 mm^−1^
*T* = 113 K
0.28 × 0.24 × 0.12 mm

#### Data collection

Rigaku Saturn CCD area-detector diffractometer
Absorption correction: multi-scan (*CrystalClear*; Rigaku/MSC, 2005 ▶) *T*_min_ = 0.920, *T*_max_ = 0.965
22515 measured reflections
6397 independent reflections
5768 reflections with *I* > 2σ(*I*)
*R*_int_ = 0.036

#### Refinement

*R*[*F* ^2^ > 2σ(*F* ^2^)] = 0.036
*wR*(*F* ^2^) = 0.079
*S* = 1.01
6397 reflections
374 parameters
H-atom parameters constrained
Δρ_max_ = 0.18 e Å^−3^
Δρ_min_ = −0.34 e Å^−3^
Absolute structure: Flack (1983 ▶), 2800 Friedel pairs
Flack parameter: 0.04 (4)

### 

Data collection: *CrystalClear* (Rigaku/MSC, 2005 ▶); cell refinement: *CrystalClear*; data reduction: *CrystalClear*; program(s) used to solve structure: *SHELXS97* (Sheldrick, 2008 ▶); program(s) used to refine structure: *SHELXL97* (Sheldrick, 2008 ▶); molecular graphics: *SHELXTL* (Sheldrick, 2008 ▶); software used to prepare material for publication: *SHELXTL*.

## Supplementary Material

Crystal structure: contains datablocks I, global. DOI: 10.1107/S1600536809050612/vm2011sup1.cif

Structure factors: contains datablocks I. DOI: 10.1107/S1600536809050612/vm2011Isup2.hkl

Additional supplementary materials:  crystallographic information; 3D view; checkCIF report

## Figures and Tables

**Fig. 1.**
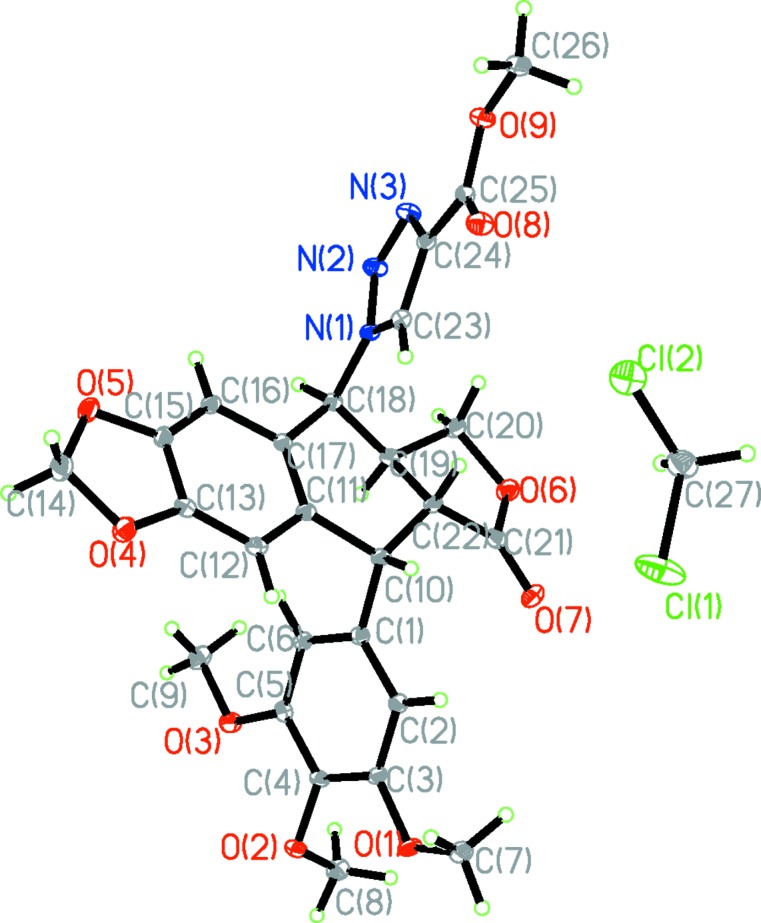
The molecular structure of (I), with atom labels and 50% probability displacement ellipsoids for non-H atoms.

## supplementary crystallographic information

### Comment

Podophyllotoxin derivatives such as Etoposide and Teniposide are in clinical use as antineoplastic agents. NK611, as well as NPF, GL-311 and TOP53 are presently under clinical trials. These podophyllotoxin ligands block the catalytic activity of DNA-topoisomerase II by stabilizing a cleavable enzyme DNA complex in which the DNA is cleaved and covalently linked to the enzyme (Hainsworth,1985). Its high toxicity has limited its application as a drug in cancer chemotherapy. Hence, extensive structure modifications have been performed since the 1950's. Meanwhile, click chemistry with copper-catalyzed Huisgen 1,3-dipolar azide-alkyne cycloaddition (CuAAC) producing 1,2,3-triazoles has exhibited increasing importance in organic chemistry due to the chemoselective nature and mild reaction conditions (Bräse, 2005). Moreover, as a functional group the 1,2,3-triazole ring has found widespread occurrence in a variety of fields. These advantages have stimulated us to couple the podophyllotoxin parent nucleus with 1,2,3-triazole to give potential anticancer candidates. Herein we report the crystal structure of the title compound (Fig.1), which can be used as a candidate for anti-tumor molecules. The asymmetric unit consists of the organic molecule and one dichloromethane molecule, C_26_H_25_N_3_O_9_.CH_2_Cl_2_. The configuration of this derivative is the same as found for podophyllotoxin, which is compatible with the mild reaction conditions. The compound contains three planar moieties: plane A consisting of atoms C_10_ to C_22_, plane B consisting of atoms C_1_ to C_6_, and the triazole ring as plane C. The dihedral angles between the planes A/B, B/C and A/C are 83.82 (6)°, 32.06 (8)° and 86.54 (7)°, respectively.

### Experimental

The title compound was synthesized from natural product podophyllotoxin by copper-catalyzed Huisgen 1, 3-dipolar azide-alkyne cycloaddition. Crystals of the title compound suitable for crystal structure analysis were obtained from a dichloromethane solution by slowly evaporating the solvent.

### Refinement

All H atoms were located in difference Fourier maps and refined independently with isotropic displacement parameters.

### Crystal data

**Table:**

C_26_H_25_N_3_O_9_·CH_2_Cl_2_	*D*_x_ = 1.506 Mg m^−^^3^
*M**_r_* = 608.42	Melting point = 212–213 K
Orthorhombic, *P*2_1_2_1_2_1_	Mo *K*α radiation, λ = 0.71073 Å
Hall symbol: P 2ac 2ab	Cell parameters from 9219 reflections
*a* = 10.377 (2) Å	θ = 1.9–27.9°
*b* = 12.639 (3) Å	µ = 0.30 mm^−^^1^
*c* = 20.463 (4) Å	*T* = 113 K
*V* = 2683.9 (10) Å^3^	Block, colorless
*Z* = 4	0.28 × 0.24 × 0.12 mm
*F*(000) = 1264	

### Data collection

**Table:**

Rigaku Saturn CCD area-detector diffractometer	6397 independent reflections
Radiation source: rotating anode	5768 reflections with *I* > 2σ(*I*)
confocal	*R*_int_ = 0.036
Detector resolution: 7.31 pixels mm^-1^	θ_max_ = 27.9°, θ_min_ = 1.9°
ω and φ scans	*h* = −13→13
Absorption correction: multi-scan (*CrystalClear*; Rigaku/MSC, 2005)	*k* = −12→16
*T*_min_ = 0.920, *T*_max_ = 0.965	*l* = −26→26
22515 measured reflections	

### Refinement

**Table:**

Refinement on *F*^2^	Secondary atom site location: difference Fourier map
Least-squares matrix: full	Hydrogen site location: inferred from neighbouring sites
*R*[*F*^2^ > 2σ(*F*^2^)] = 0.036	H-atom parameters constrained
*wR*(*F*^2^) = 0.079	*w* = 1/[σ^2^(*F*_o_^2^) + (0.0476*P*)^2^] where *P* = (*F*_o_^2^ + 2*F*_c_^2^)/3
*S* = 1.01	(Δ/σ)_max_ = 0.001
6397 reflections	Δρ_max_ = 0.18 e Å^−^^3^
374 parameters	Δρ_min_ = −0.34 e Å^−^^3^
0 restraints	Absolute structure: Flack (1983), 2800 Friedel pairs
Primary atom site location: structure-invariant direct methods	Flack parameter: 0.04 (4)

### Special details

**Table:**

Experimental. Both Cu+ and Cu2+ can be used as catalyst while combined with respective additives.
Geometry. All e.s.d.'s (except the e.s.d. in the dihedral angle between two l.s. planes) are estimated using the full covariance matrix. The cell e.s.d.'s are taken into account individually in the estimation of e.s.d.'s in distances, angles and torsion angles; correlations between e.s.d.'s in cell parameters are only used when they are defined by crystal symmetry. An approximate (isotropic) treatment of cell e.s.d.'s is used for estimating e.s.d.'s involving l.s. planes.
Refinement. Refinement of *F*^2^ against ALL reflections. The weighted *R*-factor *wR* and goodness of fit *S* are based on *F*^2^, conventional *R*-factors *R* are based on *F*, with *F* set to zero for negative *F*^2^. The threshold expression of *F*^2^ > σ(*F*^2^) is used only for calculating *R*-factors(gt) *etc*. and is not relevant to the choice of reflections for refinement. *R*-factors based on *F*^2^ are statistically about twice as large as those based on *F*, and *R*- factors based on ALL data will be even larger.

### Fractional atomic coordinates and isotropic or equivalent isotropic displacement parameters (Å^2^)

**Table:**

	*x*	*y*	*z*	*U*_iso_*/*U*_eq_	
O1	0.81730 (11)	0.46481 (8)	−0.07929 (6)	0.0222 (2)	
O2	1.02230 (11)	0.38471 (8)	−0.13992 (5)	0.0200 (2)	
O3	1.07335 (11)	0.17818 (9)	−0.13872 (6)	0.0242 (3)	
O4	1.03010 (12)	0.07454 (9)	0.18541 (6)	0.0279 (3)	
O5	1.04059 (12)	−0.10864 (9)	0.17747 (6)	0.0263 (3)	
O6	0.49041 (12)	−0.00212 (9)	−0.11823 (6)	0.0273 (3)	
O7	0.50203 (13)	0.17197 (9)	−0.09864 (6)	0.0313 (3)	
O8	0.35597 (12)	−0.14406 (9)	0.20717 (6)	0.0254 (3)	
O9	0.30253 (11)	−0.31101 (8)	0.17946 (6)	0.0242 (3)	
N1	0.60318 (12)	−0.17930 (9)	0.04644 (6)	0.0167 (3)	
N2	0.56517 (13)	−0.28046 (10)	0.03289 (7)	0.0234 (3)	
N3	0.47699 (14)	−0.30643 (10)	0.07549 (6)	0.0226 (3)	
C1	0.78479 (14)	0.18283 (11)	−0.03338 (7)	0.0162 (3)	
C2	0.75602 (15)	0.29059 (11)	−0.03655 (7)	0.0176 (3)	
H2	0.6835	0.3180	−0.0138	0.021*	
C3	0.83415 (15)	0.35759 (11)	−0.07318 (8)	0.0172 (3)	
C4	0.93996 (15)	0.31698 (12)	−0.10722 (7)	0.0165 (3)	
C5	0.96690 (15)	0.20875 (12)	−0.10457 (7)	0.0175 (3)	
C6	0.88965 (15)	0.14181 (12)	−0.06738 (8)	0.0178 (3)	
H6	0.9085	0.0683	−0.0652	0.021*	
C7	0.72731 (16)	0.51437 (13)	−0.03645 (8)	0.0261 (4)	
H7A	0.7549	0.5042	0.0089	0.039*	
H7B	0.6419	0.4828	−0.0427	0.039*	
H7C	0.7232	0.5902	−0.0462	0.039*	
C8	0.98547 (17)	0.40051 (14)	−0.20675 (8)	0.0245 (4)	
H8A	0.9016	0.4361	−0.2084	0.037*	
H8B	0.9794	0.3319	−0.2288	0.037*	
H8C	1.0502	0.4443	−0.2287	0.037*	
C9	1.09771 (18)	0.06681 (13)	−0.14314 (9)	0.0279 (4)	
H9A	1.0224	0.0314	−0.1620	0.042*	
H9B	1.1145	0.0384	−0.0994	0.042*	
H9C	1.1729	0.0545	−0.1711	0.042*	
C10	0.70229 (15)	0.11348 (11)	0.01110 (7)	0.0165 (3)	
H10	0.6540	0.1623	0.0407	0.020*	
C11	0.78712 (15)	0.04464 (11)	0.05453 (7)	0.0161 (3)	
C12	0.86601 (15)	0.09890 (12)	0.09961 (8)	0.0192 (3)	
H12	0.8637	0.1738	0.1029	0.023*	
C13	0.94594 (15)	0.03996 (12)	0.13838 (8)	0.0188 (3)	
C14	1.09057 (17)	−0.01795 (14)	0.21149 (9)	0.0278 (4)	
H14A	1.0721	−0.0239	0.2588	0.033*	
H14B	1.1851	−0.0135	0.2056	0.033*	
C15	0.95192 (15)	−0.06919 (12)	0.13392 (8)	0.0188 (3)	
C16	0.87764 (15)	−0.12393 (12)	0.09064 (8)	0.0181 (3)	
H16	0.8828	−0.1988	0.0875	0.022*	
C17	0.79280 (14)	−0.06537 (11)	0.05070 (7)	0.0156 (3)	
C18	0.70594 (15)	−0.13007 (11)	0.00655 (7)	0.0162 (3)	
H18	0.7587	−0.1880	−0.0133	0.019*	
C19	0.65391 (15)	−0.06140 (11)	−0.04854 (8)	0.0174 (3)	
H19	0.7265	−0.0457	−0.0792	0.021*	
C20	0.53937 (17)	−0.09906 (12)	−0.08870 (8)	0.0224 (3)	
H20A	0.5667	−0.1503	−0.1226	0.027*	
H20B	0.4734	−0.1325	−0.0605	0.027*	
C21	0.52869 (16)	0.08367 (13)	−0.08212 (8)	0.0224 (3)	
C22	0.60149 (15)	0.04366 (11)	−0.02292 (7)	0.0173 (3)	
H22	0.5353	0.0258	0.0108	0.021*	
C23	0.53786 (16)	−0.14106 (12)	0.09778 (7)	0.0172 (3)	
H23	0.5456	−0.0731	0.1172	0.021*	
C24	0.45700 (15)	−0.22220 (12)	0.11611 (8)	0.0169 (3)	
C25	0.36761 (15)	−0.22017 (12)	0.17193 (8)	0.0192 (3)	
C26	0.21945 (17)	−0.31309 (14)	0.23663 (8)	0.0287 (4)	
H26A	0.1500	−0.2613	0.2312	0.043*	
H26B	0.2700	−0.2954	0.2756	0.043*	
H26C	0.1824	−0.3839	0.2416	0.043*	
Cl1	0.26269 (6)	0.39288 (4)	0.23482 (3)	0.05616 (18)	
Cl2	0.21387 (5)	0.18814 (4)	0.29584 (2)	0.03992 (13)	
C27	0.1834 (2)	0.27027 (14)	0.22719 (10)	0.0343 (4)	
H27A	0.2134	0.2342	0.1870	0.041*	
H27B	0.0895	0.2823	0.2231	0.041*	

### Atomic displacement parameters (Å^2^)

**Table:**

	*U*^11^	*U*^22^	*U*^33^	*U*^12^	*U*^13^	*U*^23^
O1	0.0274 (6)	0.0121 (5)	0.0272 (6)	0.0025 (4)	0.0052 (5)	0.0009 (4)
O2	0.0216 (6)	0.0202 (6)	0.0181 (5)	−0.0065 (4)	0.0010 (5)	0.0023 (4)
O3	0.0228 (6)	0.0206 (6)	0.0293 (6)	0.0030 (5)	0.0106 (5)	−0.0002 (5)
O4	0.0315 (7)	0.0217 (6)	0.0305 (7)	−0.0010 (5)	−0.0133 (6)	−0.0014 (5)
O5	0.0284 (6)	0.0208 (6)	0.0297 (7)	0.0024 (5)	−0.0120 (5)	0.0002 (5)
O6	0.0307 (7)	0.0246 (6)	0.0267 (6)	−0.0017 (5)	−0.0094 (5)	0.0035 (5)
O7	0.0314 (7)	0.0243 (6)	0.0381 (7)	0.0046 (5)	−0.0067 (6)	0.0088 (6)
O8	0.0291 (7)	0.0228 (6)	0.0241 (6)	−0.0017 (5)	0.0064 (5)	−0.0022 (5)
O9	0.0249 (6)	0.0238 (6)	0.0239 (6)	−0.0066 (5)	0.0036 (5)	0.0035 (5)
N1	0.0195 (6)	0.0119 (6)	0.0186 (6)	−0.0005 (5)	0.0004 (5)	−0.0004 (5)
N2	0.0275 (8)	0.0157 (7)	0.0269 (8)	−0.0054 (5)	0.0019 (6)	−0.0033 (6)
N3	0.0256 (8)	0.0194 (7)	0.0227 (7)	−0.0059 (6)	0.0025 (6)	−0.0006 (6)
C1	0.0182 (7)	0.0155 (7)	0.0149 (7)	−0.0014 (6)	−0.0009 (6)	−0.0006 (6)
C2	0.0177 (8)	0.0168 (8)	0.0184 (8)	0.0011 (6)	0.0010 (6)	−0.0016 (6)
C3	0.0212 (8)	0.0121 (7)	0.0181 (8)	0.0000 (6)	−0.0037 (6)	0.0006 (6)
C4	0.0175 (8)	0.0178 (7)	0.0141 (7)	−0.0045 (6)	−0.0001 (6)	0.0016 (6)
C5	0.0162 (8)	0.0215 (8)	0.0148 (7)	0.0009 (6)	0.0015 (6)	−0.0031 (6)
C6	0.0195 (8)	0.0156 (7)	0.0184 (8)	0.0008 (6)	−0.0006 (6)	0.0002 (6)
C7	0.0264 (9)	0.0177 (8)	0.0342 (10)	0.0026 (6)	0.0033 (8)	−0.0017 (7)
C8	0.0290 (9)	0.0268 (9)	0.0178 (8)	0.0008 (7)	0.0028 (7)	0.0030 (7)
C9	0.0315 (9)	0.0257 (9)	0.0267 (9)	0.0104 (7)	0.0078 (8)	0.0010 (7)
C10	0.0185 (8)	0.0138 (7)	0.0171 (8)	0.0009 (6)	0.0027 (6)	0.0005 (6)
C11	0.0169 (8)	0.0155 (7)	0.0158 (7)	−0.0005 (6)	0.0042 (6)	0.0007 (5)
C12	0.0212 (8)	0.0157 (8)	0.0206 (8)	−0.0015 (6)	0.0031 (6)	0.0012 (6)
C13	0.0185 (8)	0.0188 (8)	0.0192 (8)	−0.0038 (6)	0.0015 (6)	−0.0020 (6)
C14	0.0287 (9)	0.0268 (9)	0.0278 (9)	0.0036 (7)	−0.0089 (7)	−0.0029 (7)
C15	0.0176 (8)	0.0191 (8)	0.0196 (8)	0.0028 (6)	0.0007 (6)	0.0017 (6)
C16	0.0192 (8)	0.0152 (8)	0.0199 (8)	0.0012 (6)	0.0024 (6)	0.0004 (6)
C17	0.0155 (7)	0.0151 (7)	0.0162 (7)	0.0005 (5)	0.0035 (6)	0.0003 (5)
C18	0.0171 (8)	0.0153 (7)	0.0162 (7)	−0.0009 (6)	0.0036 (6)	−0.0010 (6)
C19	0.0195 (8)	0.0160 (8)	0.0169 (8)	−0.0006 (6)	0.0023 (6)	−0.0010 (6)
C20	0.0262 (9)	0.0195 (8)	0.0214 (8)	−0.0008 (7)	−0.0025 (7)	0.0023 (6)
C21	0.0186 (8)	0.0255 (9)	0.0231 (8)	−0.0017 (6)	0.0006 (7)	0.0041 (7)
C22	0.0172 (7)	0.0152 (8)	0.0194 (8)	0.0004 (6)	0.0037 (6)	0.0026 (6)
C23	0.0204 (8)	0.0164 (7)	0.0148 (8)	0.0011 (6)	0.0003 (6)	−0.0007 (6)
C24	0.0184 (8)	0.0156 (7)	0.0166 (8)	−0.0006 (6)	−0.0026 (6)	0.0014 (6)
C25	0.0169 (8)	0.0215 (8)	0.0192 (8)	−0.0009 (6)	−0.0013 (7)	0.0053 (6)
C26	0.0261 (9)	0.0301 (9)	0.0300 (9)	−0.0017 (8)	0.0072 (8)	0.0105 (8)
Cl1	0.0722 (4)	0.0437 (3)	0.0526 (3)	−0.0320 (3)	0.0287 (3)	−0.0187 (3)
Cl2	0.0490 (3)	0.0366 (3)	0.0342 (3)	0.0004 (2)	0.0064 (2)	−0.0110 (2)
C27	0.0350 (11)	0.0262 (9)	0.0417 (11)	−0.0044 (8)	0.0050 (9)	−0.0084 (8)

### Geometric parameters (Å, °)

**Table:**

O1—C3	1.3722 (18)	C9—H9A	0.9800
O1—C7	1.4258 (19)	C9—H9B	0.9800
O2—C4	1.3823 (17)	C9—H9C	0.9800
O2—C8	1.4339 (19)	C10—C11	1.524 (2)
O3—C5	1.3631 (19)	C10—C22	1.535 (2)
O3—C9	1.433 (2)	C10—H10	1.0000
O4—C13	1.3710 (19)	C11—C17	1.394 (2)
O4—C14	1.430 (2)	C11—C12	1.411 (2)
O5—C15	1.3747 (19)	C12—C13	1.368 (2)
O5—C14	1.438 (2)	C12—H12	0.9500
O6—C21	1.371 (2)	C13—C15	1.384 (2)
O6—C20	1.4575 (19)	C14—H14A	0.9900
O7—C21	1.1985 (19)	C14—H14B	0.9900
O8—C25	1.2084 (19)	C15—C16	1.363 (2)
O9—C25	1.3409 (19)	C16—C17	1.411 (2)
O9—C26	1.454 (2)	C16—H16	0.9500
N1—C23	1.340 (2)	C17—C18	1.516 (2)
N1—N2	1.3664 (18)	C18—C19	1.522 (2)
N1—C18	1.4800 (19)	C18—H18	1.0000
N2—N3	1.3057 (19)	C19—C20	1.521 (2)
N3—C24	1.367 (2)	C19—C22	1.528 (2)
C1—C6	1.392 (2)	C19—H19	1.0000
C1—C2	1.396 (2)	C20—H20A	0.9900
C1—C10	1.526 (2)	C20—H20B	0.9900
C2—C3	1.392 (2)	C21—C22	1.515 (2)
C2—H2	0.9500	C22—H22	1.0000
C3—C4	1.398 (2)	C23—C24	1.377 (2)
C4—C5	1.397 (2)	C23—H23	0.9500
C5—C6	1.392 (2)	C24—C25	1.472 (2)
C6—H6	0.9500	C26—H26A	0.9800
C7—H7A	0.9800	C26—H26B	0.9800
C7—H7B	0.9800	C26—H26C	0.9800
C7—H7C	0.9800	Cl1—C27	1.7613 (19)
C8—H8A	0.9800	Cl2—C27	1.775 (2)
C8—H8B	0.9800	C27—H27A	0.9900
C8—H8C	0.9800	C27—H27B	0.9900
			
C3—O1—C7	117.47 (12)	O4—C14—O5	108.23 (13)
C4—O2—C8	112.53 (12)	O4—C14—H14A	110.1
C5—O3—C9	116.98 (12)	O5—C14—H14A	110.1
C13—O4—C14	106.31 (12)	O4—C14—H14B	110.1
C15—O5—C14	105.43 (12)	O5—C14—H14B	110.1
C21—O6—C20	109.90 (12)	H14A—C14—H14B	108.4
C25—O9—C26	114.00 (12)	C16—C15—O5	128.01 (14)
C23—N1—N2	110.51 (12)	C16—C15—C13	121.57 (15)
C23—N1—C18	130.16 (12)	O5—C15—C13	110.42 (14)
N2—N1—C18	119.31 (12)	C15—C16—C17	117.59 (14)
N3—N2—N1	107.58 (12)	C15—C16—H16	121.2
N2—N3—C24	108.46 (12)	C17—C16—H16	121.2
C6—C1—C2	120.50 (14)	C11—C17—C16	121.13 (14)
C6—C1—C10	121.51 (13)	C11—C17—C18	123.14 (14)
C2—C1—C10	117.91 (13)	C16—C17—C18	115.68 (13)
C3—C2—C1	119.62 (14)	N1—C18—C17	109.06 (12)
C3—C2—H2	120.2	N1—C18—C19	113.12 (13)
C1—C2—H2	120.2	C17—C18—C19	110.17 (12)
O1—C3—C2	125.16 (14)	N1—C18—H18	108.1
O1—C3—C4	114.67 (14)	C17—C18—H18	108.1
C2—C3—C4	120.17 (14)	C19—C18—H18	108.1
O2—C4—C5	120.10 (14)	C20—C19—C18	119.93 (12)
O2—C4—C3	119.95 (13)	C20—C19—C22	100.32 (12)
C5—C4—C3	119.81 (13)	C18—C19—C22	111.58 (13)
O3—C5—C6	125.09 (14)	C20—C19—H19	108.1
O3—C5—C4	114.81 (13)	C18—C19—H19	108.1
C6—C5—C4	120.07 (14)	C22—C19—H19	108.1
C1—C6—C5	119.82 (14)	O6—C20—C19	103.49 (12)
C1—C6—H6	120.1	O6—C20—H20A	111.1
C5—C6—H6	120.1	C19—C20—H20A	111.1
O1—C7—H7A	109.5	O6—C20—H20B	111.1
O1—C7—H7B	109.5	C19—C20—H20B	111.1
H7A—C7—H7B	109.5	H20A—C20—H20B	109.0
O1—C7—H7C	109.5	O7—C21—O6	121.17 (15)
H7A—C7—H7C	109.5	O7—C21—C22	130.67 (15)
H7B—C7—H7C	109.5	O6—C21—C22	108.15 (13)
O2—C8—H8A	109.5	C21—C22—C19	101.14 (13)
O2—C8—H8B	109.5	C21—C22—C10	120.70 (12)
H8A—C8—H8B	109.5	C19—C22—C10	114.36 (13)
O2—C8—H8C	109.5	C21—C22—H22	106.6
H8A—C8—H8C	109.5	C19—C22—H22	106.6
H8B—C8—H8C	109.5	C10—C22—H22	106.6
O3—C9—H9A	109.5	N1—C23—C24	104.66 (13)
O3—C9—H9B	109.5	N1—C23—H23	127.7
H9A—C9—H9B	109.5	C24—C23—H23	127.7
O3—C9—H9C	109.5	N3—C24—C23	108.78 (14)
H9A—C9—H9C	109.5	N3—C24—C25	125.55 (14)
H9B—C9—H9C	109.5	C23—C24—C25	125.63 (14)
C11—C10—C1	110.59 (12)	O8—C25—O9	124.24 (15)
C11—C10—C22	109.26 (12)	O8—C25—C24	122.69 (14)
C1—C10—C22	116.22 (12)	O9—C25—C24	113.07 (14)
C11—C10—H10	106.8	O9—C26—H26A	109.5
C1—C10—H10	106.8	O9—C26—H26B	109.5
C22—C10—H10	106.8	H26A—C26—H26B	109.5
C17—C11—C12	119.80 (14)	O9—C26—H26C	109.5
C17—C11—C10	124.14 (14)	H26A—C26—H26C	109.5
C12—C11—C10	116.03 (12)	H26B—C26—H26C	109.5
C13—C12—C11	117.78 (14)	Cl1—C27—Cl2	111.18 (11)
C13—C12—H12	121.1	Cl1—C27—H27A	109.4
C11—C12—H12	121.1	Cl2—C27—H27A	109.4
C12—C13—O4	128.28 (14)	Cl1—C27—H27B	109.4
C12—C13—C15	122.11 (15)	Cl2—C27—H27B	109.4
O4—C13—C15	109.60 (14)	H27A—C27—H27B	108.0
			
C23—N1—N2—N3	−0.34 (17)	C13—C15—C16—C17	−0.7 (2)
C18—N1—N2—N3	178.32 (12)	C12—C11—C17—C16	−0.9 (2)
N1—N2—N3—C24	0.55 (17)	C10—C11—C17—C16	177.31 (13)
C6—C1—C2—C3	−1.0 (2)	C12—C11—C17—C18	176.30 (13)
C10—C1—C2—C3	175.82 (14)	C10—C11—C17—C18	−5.5 (2)
C7—O1—C3—C2	11.4 (2)	C15—C16—C17—C11	1.2 (2)
C7—O1—C3—C4	−168.31 (14)	C15—C16—C17—C18	−176.13 (14)
C1—C2—C3—O1	−178.91 (14)	C23—N1—C18—C17	37.7 (2)
C1—C2—C3—C4	0.7 (2)	N2—N1—C18—C17	−140.67 (13)
C8—O2—C4—C5	92.48 (17)	C23—N1—C18—C19	−85.29 (18)
C8—O2—C4—C3	−91.68 (17)	N2—N1—C18—C19	96.36 (15)
O1—C3—C4—O2	4.1 (2)	C11—C17—C18—N1	−103.10 (16)
C2—C3—C4—O2	−175.64 (14)	C16—C17—C18—N1	74.20 (16)
O1—C3—C4—C5	179.91 (13)	C11—C17—C18—C19	21.6 (2)
C2—C3—C4—C5	0.2 (2)	C16—C17—C18—C19	−161.08 (13)
C9—O3—C5—C6	8.2 (2)	N1—C18—C19—C20	−41.98 (18)
C9—O3—C5—C4	−173.77 (14)	C17—C18—C19—C20	−164.34 (13)
O2—C4—C5—O3	−3.2 (2)	N1—C18—C19—C22	74.75 (15)
C3—C4—C5—O3	−179.03 (14)	C17—C18—C19—C22	−47.60 (17)
O2—C4—C5—C6	174.94 (14)	C21—O6—C20—C19	−22.57 (16)
C3—C4—C5—C6	−0.9 (2)	C18—C19—C20—O6	160.39 (13)
C2—C1—C6—C5	0.3 (2)	C22—C19—C20—O6	37.97 (14)
C10—C1—C6—C5	−176.39 (14)	C20—O6—C21—O7	177.91 (16)
O3—C5—C6—C1	178.54 (14)	C20—O6—C21—C22	−3.11 (17)
C4—C5—C6—C1	0.6 (2)	O7—C21—C22—C19	−153.98 (19)
C6—C1—C10—C11	45.76 (19)	O6—C21—C22—C19	27.17 (16)
C2—C1—C10—C11	−131.05 (14)	O7—C21—C22—C10	−26.7 (3)
C6—C1—C10—C22	−79.53 (18)	O6—C21—C22—C10	154.47 (14)
C2—C1—C10—C22	103.66 (16)	C20—C19—C22—C21	−38.84 (14)
C1—C10—C11—C17	−114.25 (16)	C18—C19—C22—C21	−166.96 (12)
C22—C10—C11—C17	14.9 (2)	C20—C19—C22—C10	−170.17 (13)
C1—C10—C11—C12	63.98 (17)	C18—C19—C22—C10	61.71 (17)
C22—C10—C11—C12	−166.88 (13)	C11—C10—C22—C21	−163.16 (13)
C17—C11—C12—C13	−0.1 (2)	C1—C10—C22—C21	−37.2 (2)
C10—C11—C12—C13	−178.42 (14)	C11—C10—C22—C19	−42.11 (17)
C11—C12—C13—O4	−179.74 (15)	C1—C10—C22—C19	83.85 (16)
C11—C12—C13—C15	0.7 (2)	N2—N1—C23—C24	−0.01 (17)
C14—O4—C13—C12	−179.90 (17)	C18—N1—C23—C24	−178.48 (15)
C14—O4—C13—C15	−0.29 (18)	N2—N3—C24—C23	−0.56 (18)
C13—O4—C14—O5	0.88 (18)	N2—N3—C24—C25	−178.25 (14)
C15—O5—C14—O4	−1.12 (18)	N1—C23—C24—N3	0.34 (17)
C14—O5—C15—C16	−179.58 (17)	N1—C23—C24—C25	178.03 (14)
C14—O5—C15—C13	0.96 (18)	C26—O9—C25—O8	−3.0 (2)
C12—C13—C15—C16	−0.3 (3)	C26—O9—C25—C24	176.46 (13)
O4—C13—C15—C16	−179.94 (14)	N3—C24—C25—O8	178.28 (16)
C12—C13—C15—O5	179.20 (14)	C23—C24—C25—O8	1.0 (3)
O4—C13—C15—O5	−0.43 (19)	N3—C24—C25—O9	−1.2 (2)
O5—C15—C16—C17	179.92 (15)	C23—C24—C25—O9	−178.50 (15)
